# Deterioration of the Hanson Logboat: chemical and imaging assessment with removal of polyethylene glycol conserving agent

**DOI:** 10.1038/s41598-017-14057-w

**Published:** 2017-10-20

**Authors:** Adam P. Pinder, Ian Panter, Geoffrey D. Abbott, Brendan J. Keely

**Affiliations:** 1 Department of Chemistry, University of York, Heslington, York, YO10 5DD UK; 2 York Archaeological Trust, 47 Aldwark, York, YO1 7BX UK; 3 School of Civil Engineering and Geosciences, Drummond Building, Newcastle University, Newcastle upon Tyne, NE1 7RU UK

## Abstract

The state of preservation of wood in two samples from the Hanson Logboat, currently on display in Derby Museum and Art Gallery, was analysed using elemental analysis (EA), pyrolysis–gas chromatography/flame ionisation detection (Py-GC/FID), pyrolysis–gas chromatography/mass spectrometry (Py–GC/MS) and scanning electron microscopy (SEM). The samples were collected in 2003, after the boat had undergone conservation, and in 2011 after the condition of the boat began to deteriorate. Solvent extraction enabled removal of polyethylene glycol, with which the wood had been impregnated during conservation, allowing the degradation of the cellulose and lignin polymeric components of the woods to be assessed. Elemental compositions (C, H, N, O, S), Py–GC/FID, Py-GC/MS and SEM imaging reveal extensive degradation of the wood polymers during the eight year period since conservation.

## Introduction

## Wood Composition

The structure of cellulose, the dominant biopolymer of wood, comprises unbranched chains of D-glucose molecules linked by β(1–4) glycosidic bonds^1^, the repeat unit having the formula^2^ C_6_H_10_O_5_. The lower abundance structural variant hemicellulose has shorter chain lengths and more varied structure, differing in the nature of the sugar monomers and containing saccharide side chains^3^. In particular, the dominant monomer of the hemicellulose of softwoods (gymnosperms) differs from that of hardwoods (angiosperms); mannose in the former and xylose in the latter^4,5^. The approximate molecular formula for hemicellulose is C_5_H_10_O_5._ Lignin, the other main biopolymer of wood, is structurally distinct from cellulose, comprising phenolic monomer units that differ in the nature and positions of substituents on the aromatic rings and which are extensively cross-linked. As with hemicellulose, lignin biosynthesis exhibits a degree of phylogenic specificity. Thus, the lignins of gymnosperms contain predominantly guaiacyl subunits, whereas those of angiosperms contain both syringyl and guaiacyl subunits^6,7^. Lignin is a highly complex and heterogeneous polymer that has thus far confounded definitive characterisation of its three dimensional structure, despite the application of a wide array of analytical techniques^8^. Though it will differ slightly to the intact polymer^9^, the alkaline-soluble lignin content of angiosperm wood has an approximate molecular formula of C_31_H_34_O_11_^10^. The proportions of the three wood polymers vary in different woods; in angiosperms the approximate ranges are: cellulose, 35–50%; hemicellulose, 20–35% and lignin, 15–30% and in gymnosperms are: cellulose, 40–60%; hemicellulose, 5–15% and lignin, 25–40%^11^.

## Hanson Logboat

The Hanson Logboat was discovered in 1998 in the Hanson Gravel Pit in the village of Shardlow, Derbyshire, UK; a quarrying machine uncovered the artefact though it destroyed the stern^12^. Remarkably, the vessel, carved from the trunk of a single 300 year old oak tree (*Quercus robur*), still contained its cargo of Bromsgrove sandstone, suggesting that its sinking was not intentional^13^. Carbon dating indicates an age of 1500 BC, in the Middle Bronze Age^13^. The preservation of the boat for *c*. 3400 years was undoubtedly facilitated by the waterlogged nature of the environment in which it resided. Waterlogged environments often exhibit anaerobic conditions, retarding microbial and fungal decay of wood and enabling wooden objects to survive for thousands of years^14^. It is also noteworthy here that in highly decomposed peat, the presence of lignin reflects enhanced preservation of wood under water-logged conditions^15^. Such conditions do not always prevent deterioration of wood; biological or chemical modification can still occur^16^.

The surviving 11 metres of intact logboat was cut into sections (1 m) to enable its removal from the site and each section was conserved by York Archaeological Trust with Sections 8 and 9 being joined together^13^. The conservation process involved the replacement of water in the structure of the wood with two grades of the polymer polyethylene glycol (PEG), initially impregnating with PEG 200 (a liquid at room temperature), followed by PEG 3400 (a solid at room temperature)^13^. PEG is commonly used during the archaeological conservation of wooden artefacts recovered from waterlogged environments^17^. The polymer impregnates the wood as water, which can constitute a large proportion of the mass of the artefact, is slowly removed. The PEG acts both as a cell wall bulking agent and as a consolidant, preventing the wood from cracking and deforming. Following impregnation with PEG the boat was freeze dried to remove any remaining water.

Whilst on display at Derby Museum and Art Gallery, the Hanson Logboat began to display signs of deterioration. Parts of the wooden fabric of the boat appeared to take on a darker colour and became brittle. Owing to concerns about the condition of the boat, samples were taken for analysis. Here, we present an assessment of the chemical condition of the wood based on the abundances of the main organic elements and the integrity of the wood polymers in samples of the logboat collected eight years apart.

## Experimental

### Samples and sample preparation

Two samples of wood from the Hanson Logboat were obtained; one was taken following conservation in 2003 (HL 2003; Section 8/9) and the second in 2011 (HL 2011, Section 6), after the boat showed signs of deterioration. The latter represents a region of Section 6 that showed appreciable visual signs of deterioration compared with its condition immediately following conservation treatment. A sample of freshly cut trunk wood from English oak (*Quercus robur*) was used as a modern standard. Small subsamples were cut from each of the archaeological and modern wood samples and were frozen at −20 °C before being freeze dried at approximately 1 hPa for 2 h using a Thermo Heto PowerDry PL3000. Dried samples were ground to a fine powder with an agate pestle and mortar.

### Accelerated solvent extraction

Powdered wood samples were weighed into pre-cleaned stainless steel cells (internal volume 5 mL) and extracted using a Dionex/Thermo Scientific ASE 350 accelerated solvent extractor system. Six sequential extractions were performed at approximately 10 MPa and 100 °C for 5 min using HPLC grade solvent (9:1 v/v dichloromethane (DCM)/methanol(MeOH) × 3, and acetone × 3). The extracted wood samples were allowed to dry in air prior to analysis.

### Elemental analysis

Unextracted and extracted wood (1 to 2 mg) was weighed into tin (for carbon, hydrogen, nitrogen and sulfur) or silver (for oxygen and total organic carbon; TOC) 8 × 5 mm capsules. Samples for TOC analysis were treated with aqueous HCl (18.5% w/v, 2 drops) and heated to 80 °C for 6 min to remove inorganic carbon and remove excess HCl^18^. Weighed capsules were sealed by folding and were analysed using a Thermo Scientific Flash 2000 elemental analyser. Sulfanilamide and cysteine standards were analysed to assess the accuracy of the measured values. Each wood sample was analysed in triplicate and average elemental compositions were calculated (Table 1).

**Table 1:** Mean element atomic abundances (*n* = 3) of modern oak and wood from the two Hanson Logboat samples (conserved and museum sample) before and after accelerated solvent extraction with 9:1 DCM-methanol and acetone (Δ% represents the percentage difference in elemental composition compared with modern oak).

Wood	%TOC(Δ%)	SD	%C (Δ%)	SD	%H (Δ%)	SD	%N (Δ%)	SD	%S (Δ%)	SD	%O (Δ%)	SD
Untreated wood samples												
Modern Oak C_22_H_38_O_9_	37.1	1.9	44.8	0.4	6.3	0.1	0.2	0.1	0.0	—	22.3	0.1
Conserved wood C_20_H_44_O_8_	44.0 (18. 8)	1.9	48.5 (9.7)	1.2	7.4 (26.9)	0.1	0.41 (122)	0.4	1.5	0.2	22.1 (4.1)	0.2
Museum sample C_2_H_11_O_2_	2.2 (−94.1)	0.2	4.0 (−91.0)	1.1	1.9 (−67.5)	0.4	0.1 (−58.5)	0.1	27.4	2.4	6.1 (−71.4)	0.1
After ASE extraction												
Modern Oak C_22_H_38_O_9_	37.1	2.7	44.2	0.7	5.9	0.1	0.2	0.2	0.0	—	21.2	0.2
Conserved wood C_23_H_32_O_7_	44.9 (21.2)	1.5	46.3 (4.7)	0.0	5.4 (−7.8)	0.2	0.0	—	0.0	—	19.6 (−7.9)	0.2
Museum sample C_1_H_5_O_2_	2.1 (−94.4)	0.3	2.2 (−95.1)	0.0	0.9 (−85.2)	0.0	0.0	—	27.3	0.8	4.2 (−80.0)	0.3

### Pyrolysis–gas chromatography (Py–GC)

Powdered wood samples (*c*. 1 mg) were weighed into a quartz boat and analysed using a CDS Pyroprobe 5150 coupled to a Thermo Scientific Trace GC Ultra gas chromatograph. Samples were pyrolysed at 610 °C for 15 s under analytical grade helium flowing at 9 mL/min. The valve oven, transfer line and GC inlet were held at 310 °C. Separation of the effluent was achieved using a fused silica capillary column (DB-5, 60 m × 0.32 mm i.d., 0.25 μm film thickness), with an oven temperature program: 50 °C (5 min) to 320 °C (20 min) at a rate of 4 °C/min. Helium carrier gas was used at a flow rate of 2 mL/min. Analytes were detected using a flame ionisation detector and assigned by comparison of retention times and peak patterns with commercially available compounds known to be produced by lignin pyrolysis (Sigma Aldrich), published data and subsequently collected pyrolysis–gas chromatography/mass spectrometry (Py–GC/MS) data^19–23^. Py–GC/MS was performed using the conditions detailed above, a CDS Pyroprobe 1000 connected through a CDS 1500 valved interface to an HP 6890 gas chromatograph, coupled to an HP 5972 mass selective detector (MSD) with the following set values: electron voltage 70 eV, filament current 220 μA, source temperature 230 °C, quadrupole temperature 150 °C, multiplier voltage 2200 V and interface temperature 320 °C. The acquisition was controlled by an HP Kayak XA Chemstation computer in full scan mode (*m/z* 50–650). TIC data were assigned by comparison of mass spectra with the NIST 08 database and the online NIST Mass Spectrometry Data Center reference database^24^. FID data were used for semi quantitative analysis. No replicate analyses were performed.

### Scanning electron microscopy

Sub samples for SEM were not cleaned or dried as the treatments applied during the conservation removed water and particulate matter. The material was cut with razor blades, mounted on aluminium stubs using epoxy resin and earthed using Acheson Silver DAG glue. A layer of gold/palladium (7 nm) was applied to the mounted samples using a sputter coater. Images from the freshly cut surface were obtained under vacuum using a JEOL JSM-6490LV scanning electron microscope.

## Results and Discussion

Two samples of wood from the Hanson Logboat, one collected immediately after conservation in 2003 (HL 2003 Section 8/9, hereafter referred to as conserved) and the second in 2011 (HL 2011 Section 6, hereafter referred to as museum sample), were examined following the signs of deterioration of the boat within the museum. Although small variations in the proportions of lignin, cellulose and hemicellulose occur among trees of the same species and even with individual trees as a result of environmental stresses^25–27^ they are very limited compared with the changes that occur during wood decay^20,27^. Hence a single sample of freshly cut trunk wood from English oak (*Quercus robur*) was used as a modern standard.

Small subsamples were cut from each of the archaeological and modern wood samples for determination of their elemental compositions, biopolymer content and nature and the ultrastructural appearance of the archaeological samples.

### Elemental compositions

The TOC of the conserved wood is *c*. 7% higher than that of modern oak (Table 1), consistent with depletion of holocellulose and enrichment of the lignin component of the wood, lignin having a considerably higher proportion of carbon (57.7 ± 1.4%) than holocellulose (41.4 ± 1.6%)^28^. The elemental abundances (EA) of C, H, N and S are higher than for modern oak and the oxygen content is similar (Table 1). Thus, the calculated molecular formula for the sample is C_20_H_44_O_8_, with trace levels of N and S, whereas that for modern oak is C_22_H_38_O_8_ with trace levels of N. Enrichment in lignin *via* depletion of cellulose would be accompanied by a small decrease in hydrogen content, a larger decrease in oxygen content and an increase in the carbon content. The similarity in oxygen content to modern oak together with the higher levels of H, N and S indicate the presence of other organic matter contaminating the archaeological wood.

The significantly lower TOC content of the museum sample indicates that the sample is predominantly inorganic in composition, which signifies severe attrition of the organic components of the wood (Table 1). The elemental abundances similarly reflect the low abundance levels of organic matter. The high proportion of sulfur to carbon is inconsistent with the presence of organic sulfur, hence it is apparent that the wood contains high levels of inorganic sulfur species.

### Polymeric contents

**Figure 1:**
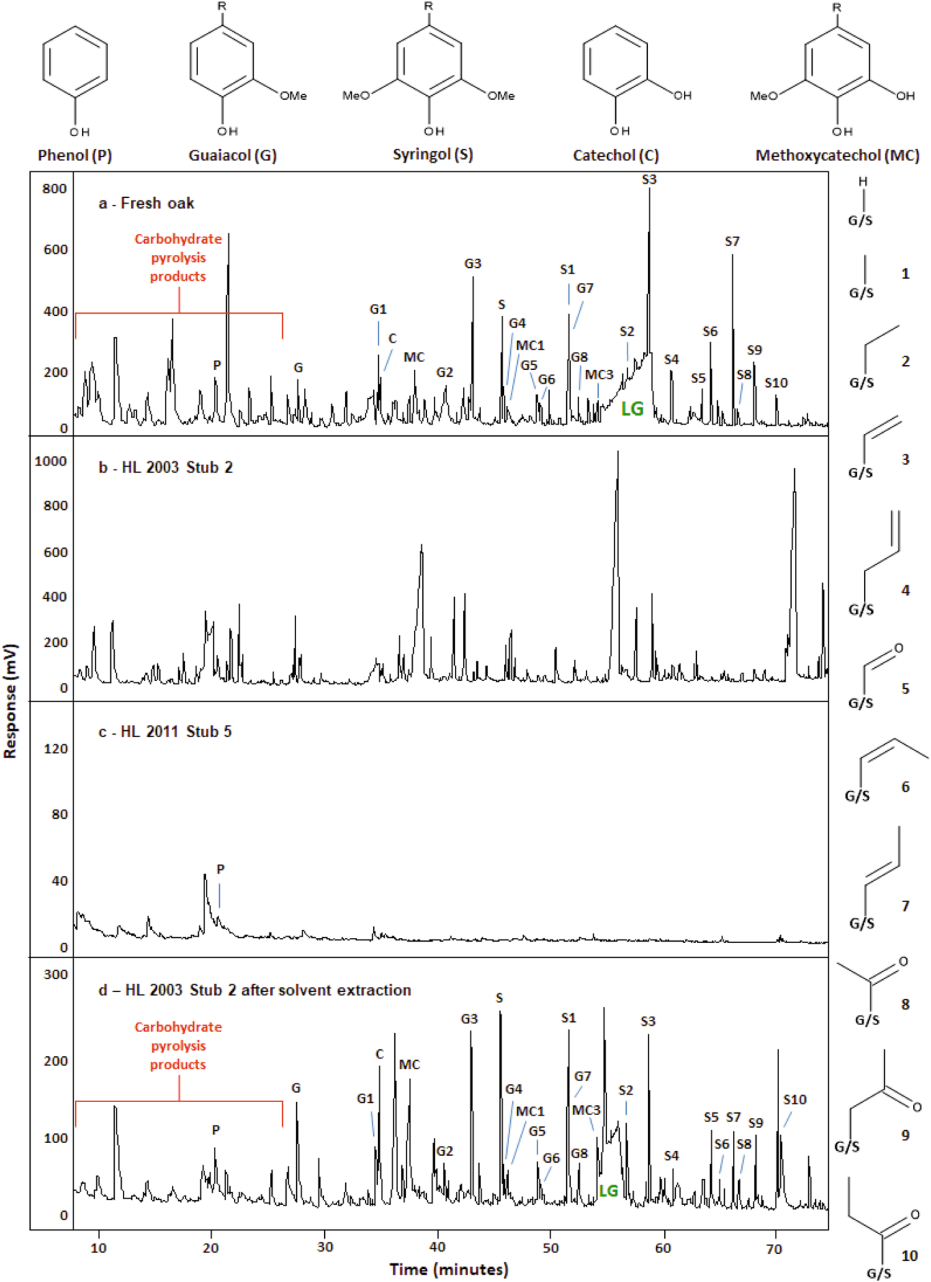
Partial Py−GC/FID pyrograms of (**a**) modern oak, (**b**) conserved wood, (**c**) museum wood sample and (**d**) conserved wood after solvent extraction. LG = levoglucosan, produced by the pyrolysis of cellulose.

Py–GC/MS was used to examine polymeric structures in the woods by liberating the constituent monomers *via* thermal cleavage. Contrasting with the profile of monomer units from the modern oak standard (Fig. 1a), which was very similar to profiles from other studies^20,22^, the pyrogram of the conserved wood reveals a sequence of regularly spaced peaks eluting between 20 and 75 min, the broadness of some of which reflect co-elution (Fig. 1b). The regularity in the elution times of the peaks in the latter is typical of a large polymer with only a few unique monomeric units. Comparison with literature data suggested that the unknown polymer was polyethylene glycol (PEG); the pyrogram of PEG exhibits very similar regularly spaced peaks^29^. Combined with the knowledge that PEG was used in the conservation of the wood, the peaks in the pyrogram from the conserved wood can be attributed to PEG. The presence of PEG compromises the interpretation of the Py–GC chromatogram as it masks key signatures from the thermal breakdown of the wood polymers. Hence, in the presence of the PEG contaminant many of the lignin-derived peaks are obscured and identification and assessment of the chemical integrity of the wood polymers cannot be performed. The presence of PEG is reflected in the elemental compositions of samples, partly explaining the atypical C, H, and O values obtained for the Hanson Logboat wood samples. PEG impregnation would not, however, account for the observed high sulfur content (the PEG used does not contain sulfur). A range of different PEG molecular weight mixtures are used in conservation of wooden objects; for example PEG 3400, commonly used as the main treatment as it penetrates into the cell walls of wood^30^, has an average formula C_2_H_4_O (which can be written as C_20_H_40_O_10_ to aid comparison with wood biopolymer formulae)^31^. The presence of PEG in the conserved wood accounts for the elevated O and H contents, which would be expected to be depleted significantly in an archaeological wood sample due to the attrition of cellulose.

The pyrogram of the museum sample (Fig. 1c) reveals very extensive degradation of both the cellulose and the lignin biopolymers. The pyrogram, representing a signal strength one order of magnitude less than for the conserved wood, was obtained from an order of magnitude more material (Fig. 1). The only identifiable peak in the pyrogram is that of phenol and no PEG was detected. These data, combined with that from EA, indicate that the material is very heavily decayed and, from a chemical perspective, is no longer recognisable as wood.

### Ultrastructural details of the wood samples

**Figure 2:**
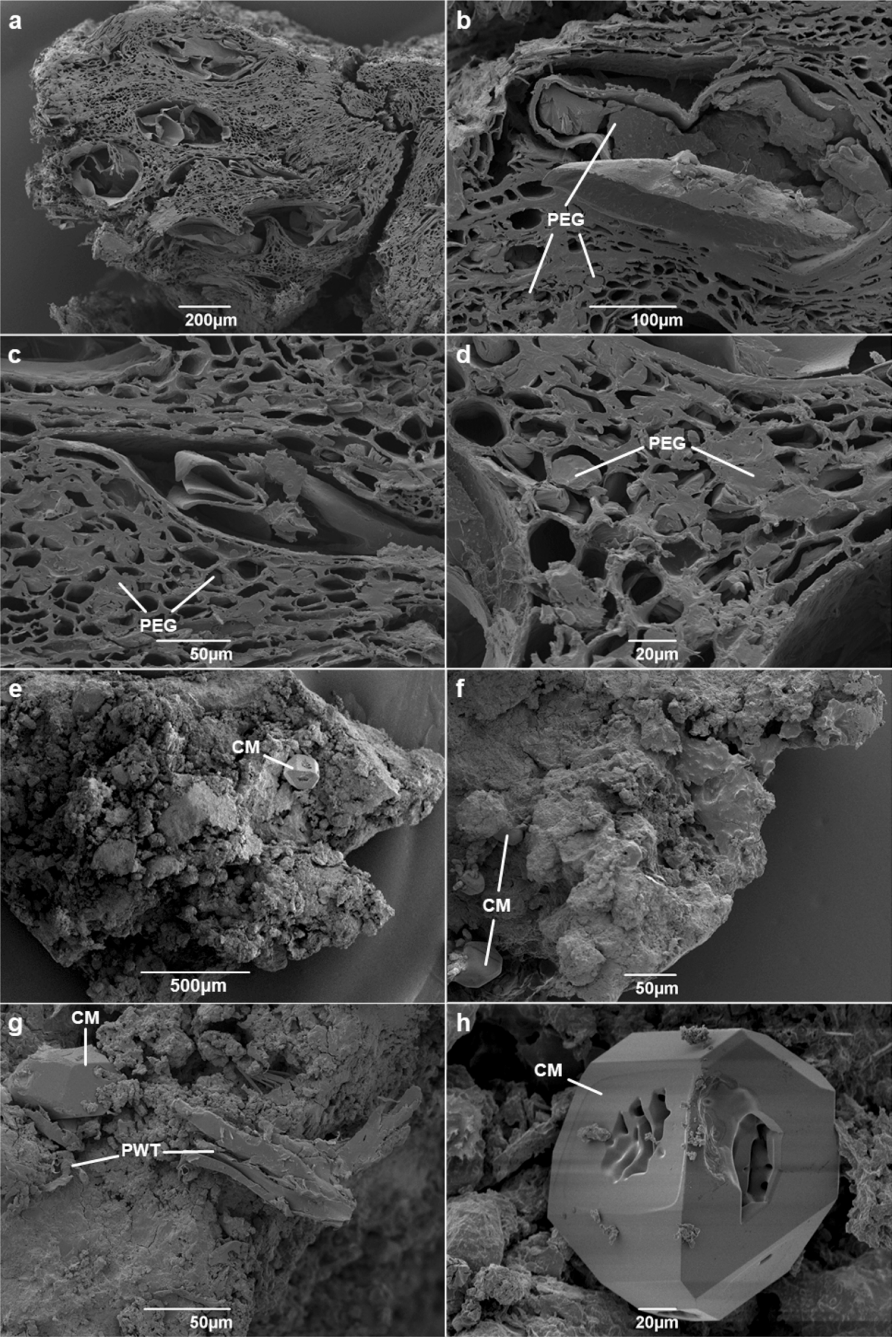
SEM images of conserved wood (**a** to **d**), showing PEG embedded in the voids of the wood substructure, and museum wood sample (**e** to **h**), showing crystalline material (CM) and probable woody tissues (PWT).

The conserved and museum samples of wood from the logboat were examined under scanning electron microscopy (SEM) to compare ultrastructural features. In the conserved wood sample PEG can clearly be seen filling many of the void spaces in the wood (labelled PEG in Fig. 2a–d). The PEG fills a large proportion of the vessels and other voids in the wood microstructure, suggesting that the conservation treatment was successful in replacing water and incorporating the PEG consolidant into the wood. There is, however, evidence of substantial distortion of the wood structure, the majority of the cells appearing compressed. This feature is often observed in waterlogged wood after it has been dried during conservation treatments^16^. Despite the evident compression the sub-structures appear relatively intact, suggesting that the incorporation of PEG was effective in preventing collapse of the dried wood. Although the cell walls appear to be thinner than those of modern oak^11,16^, they are of a thickness that suggests that secondary cell wall material (holocellulose-rich material) is reasonably well preserved. No conclusive signs of microbial attack (such as pitting or fungal hyphae) were observed, though longitudinal sections were not also examined.

The SEM image of the museum wood sample shows the structure to bear little resemblance to either the conserved wood or to modern oak, instead it has a similar appearance to mineral or soil material (Fig. 2e–h). Several features could be interpreted as being wood-like structures (labelled PWT in Fig. 2g). A crystalline inorganic mineral component is visible on the surfaces of the sample, exhibiting irregular polyhedral crystal faces of 4, 5 and 6 sided polygons (labelled as CM in Fig. 2e–h). A crystal of the mineral (approximately 2 mm in diameter) was observed to have a golden colour when viewed under scanning optical microscopy during the preparation of samples for SEM, prior to the sputter coating. The geometry, colouration and high sulfur content of the wood are consistent with the material being pyrite (FeS_2_).

### Removal of PEG from the archaeological wood samples

In an attempt to remove the PEG from the conserved wood, the material was subjected to sequential accelerated solvent extraction (ASE) with 9:1 DCM-methanol followed by acetone (see Experimental). Samples of modern oak and the museum wood were also extracted to remove any non-biopolymer materials contributing to the elemental composition. The sequential extraction procedure removed 59.7% of the mass of the conserved wood (Table 2), compared with c. 1% of the mass typically removed from modern, untreated oak by extraction with similar solvents^32,33^. Hence, PEG represented the largest proportion of the mass of the conserved wood sample. The removal of 91.7% of the solvent extractable material in the first extraction suggests that a single extraction with DCM:methanol would enable Py-GC analysis to reveal the lignin constituents. The subsequent extractions with DCM:methanol and acetone possibly improved the reliability of the EA data by removing remaining traces of PEG and other higher polarity compounds (e.g. tannins), producing a purer polymeric material for analysis by Py-GC and EA, though this was not verified experimentally.

**Table 2:** Mass and yield data for solvent extraction of conserved Hanson Logboat wood (434.7 mg).

Extract	Mass of extract/mg	% of Σ extract mass	% yield of sample mass
DCM:MeOH 1	238.0	91.7%	54.8%
DCM:MeOH 2	12.0	4.6%	2.8%
DCM:MeOH 3	4.9	1.9%	1.1%
Acetone 1	2.3	0.9%	0.5%
Acetone 2	1.3	0.5%	0.3%
Acetone 3	0.9	0.3%	0.2%
Σ DCM:MeOH	255.0	98.3%	58.7%
Σ Acetone	4.5	1.7%	1.0%
Σ all extracts	259.5	100.0%	59.7%

### Elemental compositions of solvent extracted samples

Solvent extraction of modern oak did not alter the TOC content of the wood and gave an elemental formula C_22_H_35_O_8_, with traces of N, very similar to that calculated before extraction (C_22_H_38_O_8_; Table 1), in agreement with an earlier study^22^. The extracted conserved wood sample gave a marginally higher TOC content than before extraction. The calculated molecular formula, C_23_H_32_O_7_, differs markedly from the conserved wood before extraction. The formula gives a closer match to the extracted oak than before extraction. Thus, the extracted conserved wood has a higher carbon content and lower hydrogen and oxygen contents than the modern oak standard. Such differences are consistent with attrition of the holocellulose fraction, as would be expected for an archaeological wood sample^34^. Coupled with the observed differences in the TOC content before and after extraction, it is probable that a large quantity of the PEG has been removed by the extraction process, and that the elemental composition of the extracted conserved wood sample reflects only the wood component.

Sulfur was absent from the solvent extracted conserved wood sample, indicating that the sulfur in the unextracted sample represented elemental or organic sulfur species that are amenable to solvent extraction. The TOC content of the solvent extracted museum sample was essentially the same as that of the unextracted sample. Whereas the extracted museum sample had a lower carbon content than the unextracted material, the sulfur contents of the native and solvent extracted wood samples are essentially identical (Table 1). The resistance of the sulfurous material to extraction by organic solvents suggests it to comprise inorganic sulfur such as pyrite (FeS_2_).

### Polymeric contents of the extracted woods

The absence of signatures of PEG in the pyrogram of the conserved wood after solvent extraction (Fig. 1d) indicates complete removal of the polymer from the wood. Pyrograms of the solvent extracts contained abundant signatures of PEG, consistent with it being removed by the DCM:methanol solvent (data not shown). The profile of the pyrolytic breakdown products from the extracted conserved wood sample is very similar to that of modern oak (Fig. 1a), but with several key differences. The lower relative abundance of carbohydrate pyrolysis products in the pyrogram of the archaeological wood is attributable to degradation of cellulose and hemicellulose. The species eluting before 26 min are furan based aldehydes and ketones produced by thermally induced degradation reactions of 6-membered ring sugars from both cellulose and hemicellulose. As a result, the compounds cannot be assigned specifically to either polymer. A decrease in the peak area of levoglucosan, a product of the thermal degradation of cellulose, suggests that the cellulose component of the wood has certainly been degraded. The most commonly observed biological degraders of wood in waterlogged or aquatic environments are bacteria, fungi playing a lesser role than is typically observed in other burial environments^35–38^. Typical bacterial wood degraders preferentially attack the carbohydrate components, with limited modification of the lignin component^14,25,35,39^. The presence of only residual amounts of holocellulose in the conserved wood sample indicates limited preservation of the more labile component of the original wood polymer composition.

The pyrograms of modern wood and the conserved wood reveal clear differences in their lignin contents and in the abundance of phenol relative to the summed total of lignin-derived products (Table 3). Microbially mediated decay of lignin subunits typically results in a measurable decrease in the ratio of total syringyl to total guaiacol (S:G ratio)^34,40,41^. Previous studies of archaeological wood recovered from waterlogged environments has suggested that the syringyl units are selectively degraded, either by being chemically modified in-chain or involving their complete removal from the lignin polymer, leading to a lower S:G ratio than for un-degraded wood of the same species^20^. The S:G ratios of modern oak (1.62) and the conserved sample (0.94) are consistent with such degradation having affected the archaeological wood. The S:G ratio and ratios of the majority of syringyl and guaiacyl structural pairs (components having the same alkyl substituents) are lower in the conserved sample than in modern oak, suggesting that the archaeological wood has experienced preferential loss of syringyl lignin components (Table 4). This indicates that the lignin component of the Hanson Logboat was substantially altered prior to its conservation in 2003. Several mechanisms have been suggested to explain the greater susceptibility of syringyl than guaiacyl moieties in angiosperm lignin. One relates to the greater extent of cross linking that is possible with guaiacyl units, *via* both β–O4 aryl ether linkages and by carbon–carbon bonds at the C5 position of the aromatic ring. By contrast, syringyl units have a methoxyl group at C5 and so cannot form cross-linking carbon–carbon bonds. The ensuing greater degree of polymerisation of guaiacyl units potentially makes them more resistant to enzymatic attack^21,22^. Another plausible explanation is that many lignolytic microbes may preferentially attack the secondary cell wall layers that are richer in syringyl lignin, leaving the guaiacyl lignin rich inner lamella intact and hence having a greater impact on syringyl units than on guaiacyl units^42^.

**Table 3:** Semiquantitative analysis of lignin derived phenols produced during Py–GC/FID of modern oak and conserved Hanson Logboat wood. Values are expressed as percentages of the sum of all lignin phenol peak areas.

Pyrogram compound	Modern oak	Conserved Hanson Logboat
(P) Phenol	5.45	3.39
(G) Guaiacol	3.44	7.61
(G1) 4-Methylguaiacol	6.15	2.28
(C) Catechol	3.09	7.36
(MC) Methoxycatechol	6.65	11.24
(G2) 4-Ethylguaiacol	6.22	1.44
(G3) 4-Vinylguaiacol	7.94	6.18
(S) Syringol	5.56	7.15
(G4) 4-Allylguaiacol	1.97	1.37
(MC1) 4-Methylmethoxycatechol	1.73	2.00
(G5) Vanillin	1.81	2.29
(G6) *cis*-Isoeugenol	1.08	0.85
(S1) 4-Methylsyringol	4.52	5.65
(G7) *trans*-Isoeugenol	4.59	4.02
(G8) Acetoguaiacone	2.19	1.67
(S2) 4-Ethylsyringol	1.33	0.31
(MC3) 4-Vinylmethoxycatechol	1.49	9.60
(S3) 4-Vinylsyringol	9.70	5.60
(S4) 4-Allylsyringol	2.75	1.01
(S5) Syringaldehyde	2.24	2.90
(S6) *cis*-4-Propenylsyringol	4.62	3.55
(S7) *trans*-4-Propenylsyringol	8.02	2.70
(S8) Acetosyringone	1.16	1.22
(S9) Syringylacetone	4.62	2.83
(S10) Propiosyringone	1.69	5.79

**Table 4:** Ratios for corresponding syringyl, guaiacyl, phenol and methoxycatechol subunits from solvent extracted modern oak and conserved Hanson Logboat wood.

Ratio	Modern oak	Conserved Hanson Logboat
SH:GH	1.62	0.94
S1:G1	0.74	2.48
S2:G2	0.21	0.21
S3:G3	1.22	0.91
S4:G4	1.39	0.73
S5:G5	1.24	1.27
S6:G6	4.28	4.19
S7:G7	1.75	0.67
S8:G8	0.53	0.73
Σ S:G	1.13	1.09
Σ S:MC	4.68	1.69
Σ G:C	11.44	3.76

**Figure 3:**
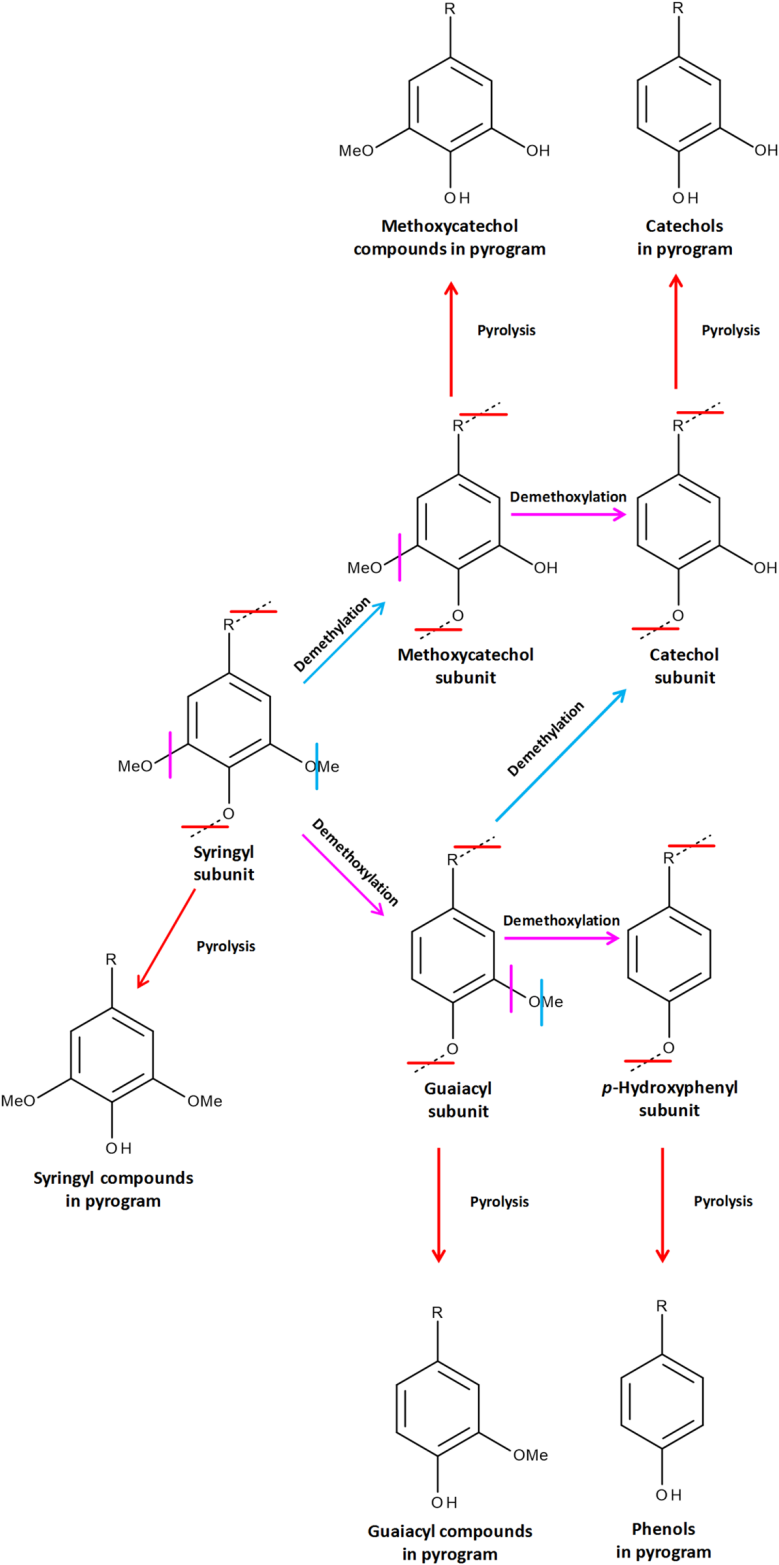
Postulated pathway for the demethoxylation and demethylation of syringyl and guaiacyl lignin subunits, leading to the formation of phenols and catechols and methoxycatechols. R = H or various alkyl substituents such as those shown in Fig. 1. Adapted from Schoemaker^66^.

Several mechanisms for the direct attrition of syringyl moieties have been proposed, the two most often postulated being demethoxylation and demethylation (Fig. 3). Demethoxylation would result in an increase in mono-methoxylated guaiacyl units, contributing to an observed decrease in the S:G ratio^40^. One of the most characteristic pyrolysis signatures of heavily degraded lignin in archaeological wood is the increase in *para*-hydroxyphenyl with respect to guaiacyl and syringyl subunits. The increase is typically a result of the complete demethoxylation of guaiacyl and syringyl subunits, leading to the liberation of large amounts of phenol on pyrolysis (Fig. 3). An increase in the amount of phenol is not evident in the Hanson logboat pyrolysis results, suggesting that complete demethoxylation of syringyl to form para-hydroxyphenyl lignin subunits, has not occurred. Demethylation of lignin phenols leads to the production of a range of benzenediol (catechol)-related compounds. The demethylation of guaiacyl units yields catechols (Fig. 3; C) whereas syringyl units can be mono-demethylated to produce methoxycatechols (Fig. 3; MC). The pyrogram of solvent extracted conserved wood reveals a marked increase in both C and MC species, the G:C and S:MC ratios both being *c*. three times smaller in the conserved wood than for modern oak (Table 4). Demethylation of lignin phenols is commonly observed as a result of the action of the laccase enzymes of fungi including brown rot fungi^43,44^. Degradation of lignin by white rot fungi, mediated by manganese peroxidase enzymes, results in cleavage of the side chains of syringyl and guaiacyl lignin units as well as oxidation of the lignin phenols to both aldehydes and acids^45^. Recent work characterising the emerging role of bacteria in lignin degradation has demonstrated that several species of soil bacteria (including species from the genera *Pseudomonas* and *Actinomycetes*) can also modify lignin by demethylation^46–48^. Low levels of methoxycatechols have also been identified in modern wood standards and suggested to result from demethylation of syringyl subunits in the earliest stages of wood diagenesis^20^. Alternatively, the methoxycatechols may be residual lignin precursors, such as 5-hydroxy-coniferaldehyde^7^. In addition, the abundance of lignin phenols with shorter side chains is higher, and the prevalence of longer side chains lower, than for modern oak. Higher levels of guaiacol, syringol, 4-methylsyringol (S1) and 4-ethylsyringol (S2) in the conserved wood suggest that some modification to the three carbon linkages between the phenolic moieties has occurred, leading to depolymerisation of the lignin. Cleavage of the Cα–Cβ linkages of the lignin polymer (generating 4-methylsyringol) and demethoxylation (producing guaiacol) can both occur following the formation of aromatic radicals induced by microbial lignolytic peroxidases^43,49^. Similar changes were reported for angiosperm wood exposed to a range of delignifying basidiomycetes^21^. Contrary to the overall low molecular weights of the conserved wood pyrolysis products, a large amount of a ketone containing component, propiosyringone (S10), was liberated. Two other oxidised lignin compounds occurring in high relative abundance are vanillin (G5) and acetosyringone (S8). Such products of oxidative modification of lignin side chains have been reported from wood treated with white rot fungi, and are also attributed to the cascade of depolymerisation reactions that follow enzymatic radical generation^21,50^. Such modifications typically occur in oxic environments. Whether this reflects degradation of the wood prior to its burial or degradation during the time between excavation and the commencement of conservation treatments is not known. Such oxidative degradation processes may be expected to have been limited *in situ* as the conditions would have rapidly become anoxic^51^.

The pyrogram of the solvent extracted museum sample showed no differences to the unextracted material (Fig. 1c), suggesting that the low amount of organic material remaining in the sample is not amenable to solvent extraction and is probably polymeric and highly resistant. The lack of peaks in the carbohydrate region of the pyrogram, and the simplicity of the peak patterns compared with those from modern oak, implies that holocellulose is absent and that any remaining lignin is in an extremely degraded form. The heavily degraded lignin has lost much of its peripheral functionality, the defunctionalised remnants producing phenol on pyrolysis (as in the final step in Fig. 3). Recent experimental work produced very similar pyrograms by treating modern angiosperm wood with sulfuric acid (pH 1 for 16 weeks at 80 °C)^52^. Inorganic sulfurous species such as pyrite have been shown to aggregate in the microstructures of wood, originating either directly from the environment or by the reaction of iron(III) oxide (Fe_2_O_3_) with dissolved sulfides (H_2_S, HS^−^ or S^2−^)^53,54^. Reduced sulfur species such as pyrite are known to oxidise in air in the presence of moisture, producing acid ferrous sulfate solutions, as shown in equation (1)^53,55,56^. Airborne moisture is attracted to conserved archaeological materials due to the hygroscopic properties of PEG and the sulfuric acid thus formed has been shown to damage archaeological woods^53,54,57^.

1 $$2{{\rm{FeS}}}_{2(s)}+7{{\rm{O}}}_{2(g)}+2{{\rm{H}}}_{2}{{\rm{O}}}_{(l)}\to 2{{\rm{Fe}}}_{(aq)}^{2+}+4{{\rm{SO}}}_{4(aq)}^{2-}+4{{\rm{H}}}_{(aq)}^{+}$$

Further work on this material, to identify the type of inorganic sulfur compounds present, could be carried out. Possible methods of analysis include X-ray diffraction (either by isolation of the sulfurous material or in conjunction with scanning electron microscopy) and sulfur and iron K-edge XANES^58^.

## Conclusions

The two logboat samples reveal very different states of preservation. The conserved wood displays some attrition of the holocellulose fraction. The lignin component has also been degraded as indicated by a low lignin S:G ratio, partial modification of the lignin by demethylation of substituted aromatic phenols and higher amounts of short chain and oxidised lignin compounds formed by oxidative depolymerisation. Degradation of celluloses and demethylation of lignin both occur early during decomposition of wood in archaeological burials, whereas depolymerisation and oxidation of the aryl three carbon linkages typically occur in oxic environments as a direct result of microbial decay. It is uncertain if the decay of the bipolymers occurred prior to burial, within the burial environment or post excavation. SEM imaging suggests that the microstructure of the wood is relatively well preserved, owing to the successful impregnation by PEG. By contrast, the museum sample is very heavily degraded, with no cellulose and very little, heavily modified, lignin remaining. The high level of inorganic sulfur-containing mineral in the material indicates a likely route to enhanced degradation of the wood through oxidative generation of sulfuric acid, lowering the pH of microenvironments within the wood. Finding a solution to the acidification of wood by sulfur compounds is a major focus of current research in the conservation community^59–63^.

A particularly novel and interesting aspect of the study is the acquisition of a comprehensive set of analyses that accurately and directly reflect the preservation state of the constituent biopolymers after removal of polyethylene glycol conservation treatments. Previous work on the analysis of woods conserved with PEG have either used solid state NMR techniques combined with data manipulation to remove resonances from PEG^58,64^ or have analysed the wood using Py–GC/MS without the removal of the PEG, relying on data subtraction and sequential pyrolysis at different temperatures to provide partial characterisation of the condition of the wood^65^. The ability to remove chemical contaminants completely and easily from materials that have undergone similar conservation processes potentially allows for the future analysis of precious artefacts in collections. This is an exciting prospect as it could facilitate the analysis and assessment of objects that hold vast cultural, social and historical significance.

### Data availability

The datasets generated during and/or analysed during the current study are available from the corresponding author on reasonable request.
